# New York Veterinary Societies

**Published:** 1894-02

**Authors:** 


### NEW YORK VETERINARY SOCIETIES.

---

Pursuant to a call from the New York State Veterinary Medical Association, most gratifying active interest is being taken by the County Secretaries throughout the State, in the formation of local societies.

Dr. Thomas Giffen, New York County, called a meeting on January 25th, which was aided by the presence of Dr. W. H. Hoskins, of Philadelphia, President of the United States Association. Thirty-seven practitioners answered his call and organized.

Dr. J. P. Thompson, Niagara Co., reports a promising list in his county.

Dr. Wilson Huff, Oneida Co., has issued a call to a large number.

Dr. J. M. Chase, Seneca Co., having a smaller number, will include adjoining counties in his organization.

Dr. Jno. A. Bell, Jefferson Co., will also include neighboring districts.

Dr. E. H. Nodyne, Wayne Co., has called a meeting for February 12th.

Dr. Genung, Thompkins Co., and other County Secretaries report progress.

